# Longitudinal PTSD trajectories before and after the October 7, 2023, terror attacks: A nationwide study of Israeli adults

**DOI:** 10.1192/j.eurpsy.2025.10130

**Published:** 2025-11-03

**Authors:** Yafit Levin, Dvora Shmulewitz, Vera Skvirsky, Merav Vider, Ariel Kor, Shauli Lev-Ran, Mario Mikulincer

**Affiliations:** 1 Social Work, Ariel University, Ariel, Israel; 2 Department of Psychology and Azrieli Israel Center for Addiction and Mental Health, The Hebrew University of Jerusalem, Israel; 3 Medicine, The Hebrew University of Jerusalem Faculty of Medicine, Israel; 4 Faculty of Medicine and Health Sciences, Tel Aviv University, Israel

**Keywords:** post-traumatic stress disorder (PTSD), pre–post trauma, trajectories

## Abstract

**Background:**

Existing research on post-traumatic stress disorder (PTSD) development over time covers brief periods predominantly among military personnel, rather than civilians, and baseline measurements from before traumatic experiences are rarely available. This longitudinal study examined PTSD trajectories among Israeli civilians before and after the October 7, 2023, terror attack, exploring their associations with current and previous trauma.

**Methods:**

Data included 1,231 Israeli Jewish adults from a quasi-representative sample surveyed at four time points: January 2018, April 2022, December 2023, and March 2024. Participants completed self-report measures, including the PTSD Checklist for DSM-5 (PCL-5), exposure to the October 7 attack and subsequent war, and trauma in childhood and adulthood. Latent growth mixture modeling identified PTSD trajectories.

**Results:**

Four trajectories were identified: resilience (70.4%), trauma recovery (9.4%), trauma vulnerability (16.8%), and chronic PTSD (3.5%). The chronic PTSD group exhibited persistently high symptoms, associated with greater childhood trauma and war-related exposure. The trauma vulnerability group showed low-medium pre-attack PTSD levels that escalated post-attack, associated with higher war-related stressors. The trauma recovery group had high pre-attack PTSD severity related to high trauma exposure, but showed significant symptom reduction post-attack.

**Discussion:**

This study, the first to examine PTSD trajectories in civilians after large-scale trauma, highlights diverse impacts. Most participants demonstrated resilience, while some exhibited chronic symptoms. Two trajectories – trauma vulnerability and trauma recovery – were event-responsive, suggesting that collective trauma can both exacerbate and paradoxically alleviate symptoms. Findings emphasize the need for targeted interventions and suggest future research using machine learning to refine PTSD trajectory prediction.

## Introduction

Post-traumatic stress disorder (PTSD) is a debilitating condition often arising after traumatic events like terror attacks involving targeted and indiscriminate violence [1]. It can cause intrusive memories, emotional dysregulation, and functional impairments, impacting individual well-being and societal stability. While some recover after an acute stress response [2], others develop chronic symptoms, delayed onset, or minimal reactions, reflecting variability in PTSD trajectories [2, 3]. Studying these trajectories is crucial for identifying risk factors, particularly after large-scale collective traumas like the October 7, 2023, Hamas attack – the deadliest terror incident globally in recent decades, per capita. This collective national trauma affected all Israeli citizens. PTSD prevalence rose from 16.2% six weeks pre-attack to 29.8% one month post-attack, illustrating its acute psychological impact, though the long-term toll remains unknown [4].

Studies have examined PTSD responses following terror attacks in civilians or combat in military populations. The modal outcomes show consistently identified trajectories: the resilience trajectory was almost always the majority response. The next most prevalent trajectory, recovery, was characterized by initially elevated symptoms that steadily abated, followed by a chronic high-symptom trajectory and a delayed symptom increase trajectory [5], suggesting broad replication across studies. Most longitudinal PTSD studies rely on short-term assessments or retrospective reporting [5], focus on military personnel and lack pre-trauma data, limiting insights into PTSD shifts before and after trauma, particularly in civilian populations [3, 6, 7].

Few studies have examined PTSD trajectories spanning the pre- and post-attack period [8]. For example, one study tracked college women before and after a campus shooting, finding 61% showed resilience, 29% recovered with PTSD subsiding within six months, 8% recovered gradually, and 2% had chronic symptoms [9]. No research has explored PTSD trajectories in general populations following major terror attacks and subsequent wars, leaving a gap in understanding pre- to post-trauma PTSD development. Identical post-trauma PTSD levels may stem from different trajectories – for instance, one individual may experience a trauma-related increase, while another has pre-existing high levels. Similarly, low PTSD could indicate pre-existing low levels or a decline. One study on grief and depression identified four patterns: resilience (low depression), vulnerability-to-loss (low pre-loss depression with temporary or persistent increases), chronic depression (elevated depression), and improvement (recovery from high pre-loss depression) [10].

The lack of prospective data limits understanding of factors driving variability in trauma responses, such as the interplay between pre-existing vulnerabilities (e.g., childhood and adulthood trauma) and acute, large-scale events. Studies show that traumatic experiences in childhood or adulthood, low social or educational status, and pre-existing psychiatric comorbidities predict worse post-attack outcomes [5, 11]. However, no study has distinguished between post-acute chronic PTSD and pre-to-post chronic PTSD, especially when considering pre-trauma levels from earlier trauma. Cumulative stressors like childhood trauma and adverse life events disrupt development, increase vulnerability, and reduce resilience to collective trauma [12–15]. Continuous media coverage amplifies stress responses, with graphic broadcasts heightening fear, helplessness, acute stress, PTSD, and functional impairment [15–18]. Media exposure predicts greater distress, which in turn predicts increased media use, as observed after 9/11 [19] and the Boston Marathon bombings [18]. Including pre-trauma data is critical for generating PTSD trajectories and identifying predictors. The ongoing Israel-Gaza war (October 7, 2023) provides a real-time context to study how cumulative stress and war exposure shape PTSD trajectories, including resilience, vulnerability, recovery, and chronicity.

This study assessed PTSD symptoms before and after the October 7, 2023 attack in a general population sample of Israeli Jewish adults (*N* = 1,231). Data were collected at four time points: twice pre-attack (2018, 2022) and twice post-attack (December 2023, March 2024). The study aims to: [1] evaluate changes in PTSD severity at two- and five-months post-attack compared to pre-trauma levels; [2] identify PTSD trajectories across pre- and post-trauma periods and classify respondents accordingly, examining whether these trajectories align with those observed after other traumatic events (e.g., 2,5); and [3] explore how exposure to the attack, the subsequent war, and prior trauma in childhood or adulthood relate to these trajectories. This approach provides insights into collective trauma’s long-term mental health effects.

## Method

### Participants

Cross-sectional data were collected at four time points – January 2018, April 2022, December 2023, and March 2024 – from a quasi-representative sample of Jewish adults (ages 18–70) in Israel [20]. Participants were recruited via iPanel (www.ipanel.co.il/en/) and restricted to Jewish, Hebrew-speaking individuals to avoid cultural adaptations and account for lower survey participation among older adults. Sampling in 2018, 2022, and 2023 was stratified with quotas matching key sociodemographics (gender, age, religiosity, education, residence) based on Israel Census Bureau data, ensuring a 3% margin of error [21]. In 2018, random panel members were invited, while in 2022 and 2023, previous and new respondents were screened until quotas were met. All 2023 participants were re-invited for the March 2024 survey.

In January 2018 (T1), 4,035 participants completed the survey; 2,659 in April 2022 (T2); 4,002 in December 2023 (T3); and 2,768 in March 2024 (T4). This study includes participants who participated in at least one pre-trauma wave (T1 or T2) and both post-trauma waves (T3 and T4), resulting in a sample of 1,231 participants (590 women, 47.9%); of those, 569 completed all four waves and 662 completed three. Sociodemographic details are in Table SM1 of the Supplementary Materials.

### Procedure

The authors assert that all procedures contributing to this work comply with the ethical standards of the relevant national and institutional committees on human experimentation and with the Helsinki Declaration of 1975, as revised in 2008. Responses were confidential, with no identifying information accessible to researchers or the survey company. The methodology followed the ICC/ESOMAR International Code (iPanel). All participants provided electronic informed consent prior to participation. The Institutional Review Board of the Reichman University approved this study (RIRB 1855100). Surveys were conducted via Qualtrics, and participants received 20 ILS gift cards. Quality assurance included inviting registered individuals, attention checks, and excluding inconsistent responses.

### Measures

#### PTSD symptoms

PTSD symptoms were assessed across four waves (T1-T4) using the 20-item Posttraumatic Stress Disorder Checklist DSM-5 (PCL-5; [22]), covering intrusions, avoidance, negative cognitions/mood, and arousal/reactivity. Participants rated symptoms experienced in the past month on a 5-point scale (0 = not at all to 4 = extremely). At T1 and T2, items referred to the most distressing experienced event, while at T3 and T4, they focused on the October 7 attack and the war. Total scores (range: 0–80) reflected overall PTSD severity, with excellent reliability (Cronbach’s *α* = .96) at all waves.

#### War-related exposure

At T3, participants completed eight items assessing exposure to the October 7 attack and war-related stressors, including sirens, explosions, personal or family danger, uncensored media, hate speech, and financial insecurity. Participants rated these on a 7-point scale (1 = not at all to 7 = multiple times a day). Total exposure scores (8–56; Cronbach’s α = .83) were calculated by summing the items, with higher scores reflecting greater exposure.

#### History of traumatic events during childhood

We adapted the WHO ACE-IQ [23] at T3 to assess 11 ACEs across two domains: childhood maltreatment and household dysfunction. Childhood maltreatment included six items (1 = not at all to 4 = a lot): one each for physical neglect, physical abuse, emotional neglect, and emotional abuse, and two for sexual abuse (scores: 1–24, M = 6.68, SD = 2.46). Household dysfunction included five yes/no items: parental substance abuse, separation/divorce, mental illness, incarceration, and domestic violence (scores: 0–5, M = 0.50, SD = 0.88). The two scores were moderately correlated (*r* = .43, *p* < .001).

#### Traumatic life events in adulthood

At T2, participants completed the 12-item List of Threatening Experiences (LTE; 24), assessing major stressful events in the past six months with yes/no responses. Recent stressful events (range: 0–10, M = 1.20, SD = 1.43) were calculated by summing affirmative responses.

#### Sociodemographic variables

Age, gender, marital status, education, and economic status were measured in T3.

### Analytic strategy

The Little MCAR test suggested data were not missing completely at random across all time points (*χ*^2^ [24] = 169.54, *p* < .0001) but were missing at random for T3 and T4 (*χ*^2^ [6] = 3.73, *p* = .714). *T*-tests showed no significant differences between valid and missing data or between participants with four versus three measurements. Thus, analyses included participants with at least three measurements. Analyses were conducted using Mplus Version 8.2 [25] with robust maximum likelihood (MLR; [26]).

To analyze overall PTSD symptom changes across waves (pre- and post-attack), a repeated measures analysis of variance (ANOVA) was performed, followed by pairwise contrasts (and absolute mean difference for effect size) to identify significant changes over time. Latent growth mixture modeling (LGMM) was used to identify profiles with distinct PTSD trajectories from T1 to T4. Models with 1–5 classes were evaluated using fit indices Akaike Information Criterion (AIC), Bayesian Information Criterion (BIC), Sample-Size Adjusted Bayesian Information Criterion (SSBIC), entropy, Lo–Mendell–Rubin Likelihood Ratio Test (LMR-LRT), and Bootstrap Likelihood Ratio Test (BLRT). The time score estimates were determined based on years since T1 (0, 4.25, 5.92, 6.17) to get a more accurate representation of the temporal pattern. The optimal model was selected based on fit indices, theoretical soundness, and parsimony [27] (Table 1). Class selection was based on (a) the lowest BIC, sample-size-adjusted BIC, and AIC; (b) significant LMR-LRT and BLRT tests; and (c) high latent class membership probabilities (entropy ≈ 0.80). A quadratic factor tested non-linear changes, evaluated beyond the linear slope for model fit improvement. Standard errors (SE) were reported for slopes, with smaller SEs indicating greater precision. Participants were assigned to the most probable class in the four-class solution. ANOVA examined trajectory differences in demographics, trauma exposure, and historical trauma. Group means reflected severity differences. Multinomial logistic regression identified PTSD class predictors (e.g., trauma-vulnerability, trauma recovery, chronic PTSD) relative to resilience and compared chronic PTSD to trauma-vulnerability trajectories, controlling for demographics (age, sex, economic status). Predictors included demographics, childhood/adulthood trauma, and current war-related stressors. Odds ratios (ORs) with 95% confidence intervals (CIs) and significance were reported (*p* < .05).

**Table 1.** Fit indices for latent growth mixture models for continuous PTSD assessment among the Israeli population

Clusters	AIC	BIC	SSBIC	Entropy	LMR-LRT *p*-value	BLRT *p* value
1	110,098.75	110,098.75	110,148.53	–	–	–
2	107,728.11	107,847.09	107,793.07	0.85	*p* < .001	*p* < .001
3	106,852.23	106,999.22	106,932.48	0.82	*p* < .001	*p* < .001
4	106,133.68	106,308.66	106,229.21	0.80	*p* < .001	*p* < .001
5	106,140.22	106,411.10	106,325.11	0.70	1.00	1.00

*Notes*: Fit indices: Akaike Information Criterion (AIC), Bayesian Information Criterion (BIC), Sample-Size Adjusted Bayesian Information Criterion (SSBIC), entropy, Lo–Mendell–Rubin Likelihood Ratio Test (LMR-LRT) and Bootstrap Likelihood Ratio Test (BLRT) test. The optimal number of classes was chosen based on (a) the lowest BIC, sample size-adjusted BIC, and AIC scores; (b) significant LMR-LRT and BLRT tests; and (c) high latent class membership probabilities, as indicated by an entropy value.

## Results

### PTSD changes after the October 7 attack

**Figure 1.**
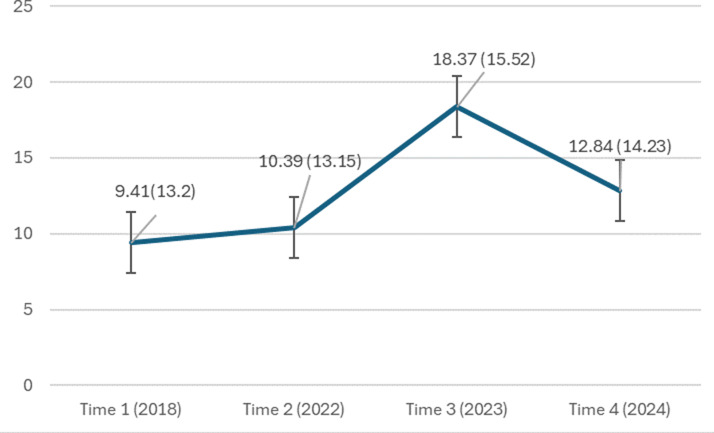
Change in PTSD severity over time in the entire sample. PTSD symptoms were assessed using the 20-item Posttraumatic Stress Disorder Checklist DSM-5 (PCL-5). Statistical significance between timepoints: T1 versus T2: *p* = .058 (ns); T1 versus T3: *p* < .001; T2 versus T3: *p* < .001; T1 versus T4: *p* < .001; T2 versus T4: *p* < .001; T3 versus T4: *p* < .001. Error bars represent standard error.

Means and standard deviations of PTSD severity across the four waves are shown in Figure 1. A repeated measures ANOVA found significant differences in PTSD severity (*F*(3, 563) = 5.03, *p* < .001). Pairwise contrasts showed no significant difference between pre-attack waves (T1 vs. T2, mean difference = 0.99, *p* = .058), but a significant increase two months post-attack (T1 vs. T3, mean difference = 8.93, *p* < .001; T2 vs. T3, mean difference = 7.95, *p* < .001). This increase persisted five months post-attack (T1 vs. T4, mean difference = 3.48, *p* < .001, T2 vs. T4, mean difference = 2.49, *p* < .001), though PTSD severity significantly declined from T3 to T4 (mean difference = 5.45, *p* < .001). These findings indicate a sharp rise in PTSD severity post-attack, followed by a mild decline three months later.

### PTSD trajectories

In the LGMM analysis, we evaluated one- to five-class (trajectory) solutions. Fit indices decreased from one to four classes but increased with five, supporting the four-trajectory solution as optimal. Significant LMR-LRT and BLRT tests further confirmed the four-class solution. The five-trajectory solution produced two small groups that merged into existing trajectories, reinforcing the four-class model. A quadratic factor was added to test for curvilinear patterns, and its significance showed that the quadratic slope provided additional explanatory power beyond linear changes over time.

**Figure 2.**
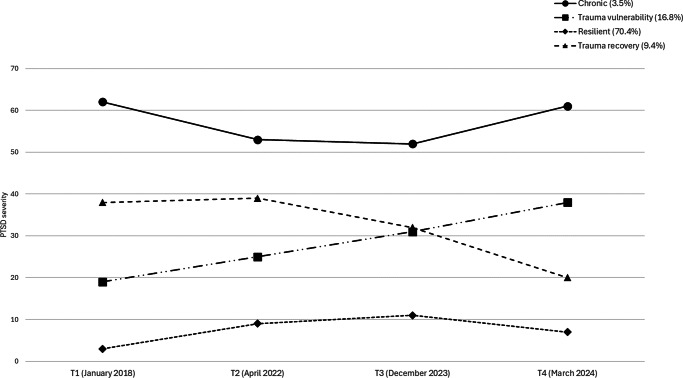
Estimated means of the four-trajectory solution across T1 to T4.

Figure 2 shows the probabilities of the four PTSD trajectories. Two trajectories – resilience and chronic PTSD – reflect little impact of the October 7 attack on PTSD severity. The **resilience** trajectory (70.4% of the sample) had a low initial PTSD severity (estimate = 3.47, S.E. = 0.16, *p* < .001) and moderate linear (estimate = 8.98, S.E. = 0.43, *p* < .001) and quadratic (estimate = −2.49, S.E. = 0.15, *p* < .001) slopes, indicating consistently low PTSD levels with a slight increase at T3 (two months post-trauma) and a slight decrease at T4 (three months later). The **chronic** PTSD trajectory (3.5% of the sample) had high initial PTSD severity (estimate = 61.41, S.E. = 2.51, *p* < .001) with linear (estimate = −13.40, S.E. = 4.90, *p* < .001) and quadratic (estimate = 4.20, S.E. = 1.73, *p* < .001) slopes, showing persistently high PTSD levels, a slight decrease from T1 to T2, and a subsequent slight increase from T2 to T4. These two groups remained largely unaffected by the October 7 attack, maintaining low or high PTSD levels before and after the trauma.

Two trajectories show a strong impact of the October 7 attack on PTSD severity: trauma vulnerability and trauma recovery. The **trauma vulnerability** trajectory (16.8% of the sample) had low-to-moderate PTSD levels at T1 and T2, increasing after the attack (T3) and further at T4 (intercept = 19.53, S.E. = 0.89, linear slope = 5.04, S.E. = 2.52, *p* < .001, quadratic slope = 0.46, S.E. = 0.92, *p* = .401). The **trauma recovery** trajectory (9.4% of the sample) started with high PTSD severity (intercept = 38.02, S.E. = 1.66, *p* < .001) and showed a significant quadratic slope (estimate = −2.98, S.E. = 0.80, *p* < .001) but a non-significant linear slope (estimate = 3.32, S.E. = 2.66, *p* = .60). PTSD severity in this group decreased slightly after the attack (T3) and more sharply at T4. Thus, 26.2% of participants were affected by the attack, with some experiencing a dramatic increase in PTSD and others showing symptom reduction.

### Classes group differences in trauma variables

War-related trauma exposure varied by PTSD trajectory (Table 2). Trauma-vulnerability, chronic PTSD, and trauma recovery groups reported higher war-related stress than the resilience group. Chronic PTSD participants had the highest childhood abuse and stress exposure, while trauma recovery participants reported more childhood trauma than the resilience group. Chronic PTSD participants also experienced the most adult trauma at T2, followed by trauma recovery, trauma vulnerability, and resilience groups (Table 2).

**Table 2.** Descriptive statistics (means and SDs) and *F*-ratios of study variables according to PTSD trajectories

Measure	Trauma vulnerability trajectory	Chronic PTSD trajectory	Resilience trajectory	Trauma recovery trajectory	*F* (3, 1227)
Trauma exposure	31.80 (9.834)^a^	32.27 (12.714)^a^	24.52 (9.301)^b^	30.19 (10.764)^a^	10.05***
Childhood maltreatment	7.54 (2.96)^b^	9.38 (3.20)^a^	6.34 (2.14)^b^	7.46 (2.90)^b^	29.52***
Childhood household dysfunction	.78 (1.13)^b^	1.38 (1.39)^a^	.40 (.76)^b^	.68 (.98)^b^	21.59***
Traumatic events during adulthood	1.55 (1.71)^a^	3.08 (2.50)^b^	.99 (1.21)^c^	2.00 (1.62)^d^	38.18***
Age	41.67 (13.91)^a^	42.23 (14.83)^a^	46.83 (13.34)^b^	45.05 (13.56)^b^	8.31***
Economic status	3.67 (1.32)^b^	2.85 (1.69)^a^	4.04 (1.37)^b^	3.87 (1.64)^b^	9.21***
Education	4.40 (1.11)	4.54 (1.21)	4.55 (1.23)	4.55 (1.19)	.754, *p* = .520
					χ^2^ (3)
Sex (women %)	51.1	42.3	52.3	54.2	1.28, *p* = .733
Marital Status (married %)	52.7	26.9	68.3	53.3	38.41***

*Notes*: *** *p* < .001. Each cell presents means (SDs). Means with different lowecase letters are significantly different at *p* < .01

### Overall model predicting PTSD trajectories

Correlations between study variables indicate significant correlations between all four measures of PTSD and all exposure factors (Supplementary Table SM2). PTSD trajectory groups had similar gender proportions and the same education levels but differed significantly by age, economic, and marital status (Table 2). Participants in the chronic PTSD and trauma vulnerability trajectories were younger than those in the resilience and trauma recovery trajectories. Relationship status also varied, with fewer chronic PTSD participants in a couple relationship (26.9%) compared to resilience participants (68.3%). Additionally, resilience participants were more likely to be in a couple relationship (68.3%) than those in the trauma vulnerability (52.7%) and trauma recovery (53.3%) trajectories. Significant group differences occurred for economic status. Resilient participants had better economic status compared to the trauma vulnerability and chronic PTSD participants. The chronic PTSD participants had the lowest income compared to all other groups.

We included age, gender, and economic status as covariates in a multinomial logistic regression to assess the contributions of trauma exposure, childhood abuse and stress, and adult traumatic events to PTSD trajectories, comparing chronic PTSD, trauma vulnerability, and trauma recovery to resilience. As shown in Table 3, the trauma vulnerability trajectory was predicted by childhood abuse, stress, and current trauma exposure. Chronic PTSD was predicted by childhood abuse and adult traumatic events, but not current trauma exposure. Trauma recovery was predicted by childhood abuse, adult traumatic events, and current trauma exposure. These findings underscore the impact of both trauma exposure and trauma history on PTSD trajectories in collective trauma contexts.

**Table 3.** Multinomial regression predicting trajectories of PTSD by pre- and concurrent-exposure factors (in reference to the resilient trajectory)

Reference group: Resilience trajectory	B	Std. error	Wald	Sig.	Exp(B)	95% CLI)	
						Lower bound	Upper bound
Trauma vulnerability trajectory							
Age	−.005	.011	.243	.622	.995	.974	1.016
Sex (female)	−.112	.251	.197	.657	.894	.546	1.464
Economic status	−.077	.091	.712	.399	.926	.775	1.107
Trauma exposure	.044***	.010	19.824	.000	1.045	1.025	1.066
Childhood maltreatment	.114***	.038	8.738	.003	1.120	1.039	1.208
Childhood household dysfunction	.248*	.108	5.334	.021	1.282	1.038	1.583
Adulthood traumatic events	.186**	.067	7.772	.005	1.204	1.057	1.372
Chronic PTSD trajectory							
Age	.001	.028	.001	.964	1.001	.948	1.057
Sex (female)	.706	.662	1.136	.286	2.025	.553	7.413
Economic status	−.192	.214	.801	.371	.826	.542	1.256
Trauma exposure	.045	.024	3.483	.062	1.046	0.998	1.096
Childhood maltreatment	.268***	.077	11.970	.001	1.307	1.123	1.521
Childhood household dysfunction	.190	.217	0.770	.380	1.209	.791	1.849
Adulthood traumatic events	.541***	.122	19.638	.000	1.718	1.352	2.182
Trauma recovery trajectory							
Age	.003	.012	.069	.793	1.003	.980	1.027
Sex (female)	−.102	.286	.126	.722	.903	.516	1.582
Economic status	.014	.105	.020	.892	1.014	.826	1.245
Trauma exposure	.042***	.012	11.603	.001	1.043	1.018	1.069
Childhood maltreatment	.101*	.049	4.297	.038	1.106	1.006	1.217
Childhood household dysfunction	.019	.144	.017	.895	1.019	.768	1.352
Adulthood traumatic life events	.357***	.077	21.777	.000	1.429	1.230	1.660

*Notes*: Multinomial regression controlling age, sex, and economic status.

## Discussion

This study offers the first comprehensive analysis of PTSD trajectories in civilians exposed to a large-scale terrorist attack, using pre- and post-trauma data. Before and after the October 7, 2023, attack on Israel, four trajectories were identified: resilience, chronic PTSD, trauma vulnerability, and trauma recovery, highlighting diverse PTSD responses. Childhood abuse and stress were prominent in the chronic PTSD group, while trauma vulnerability and recovery groups were associated with higher exposure to war-related stressors. Additionally, the trauma-recovery trajectory was related to high childhood and adult trauma levels.

### PTSD trajectories and their implications

#### Resilience trajectory

Most participants (70.4%) maintained consistently low PTSD severity over six years, demonstrating psychological stability despite the distressing events. These findings align with research showing resilience as the most common trauma outcome, explained via the assumption that resilient individuals have more adaptive strategies and stronger social networks [2, 5], which should be tested in future studies.

#### Chronic PTSD trajectory

A small subset (3.5%) showed persistently high PTSD severity, strongly linked to childhood abuse, stress, and prior adult trauma, highlighting the impact of cumulative trauma across the lifespan on enduring PTSD [28]. These findings emphasize the need for early interventions for individuals with extensive trauma histories to prevent chronic distress.

#### Trauma vulnerability trajectory

Approximately 16.8% of participants showed a sharp increase in PTSD severity after the October 7 attack, reflecting heightened trauma sensitivity. This group reported greater exposure to war-related stressors and accumulated adult trauma compared to resilient individuals, aligning with patterns seen in populations facing prolonged stressors like war or media overexposure [29–31]. These findings support diathesis-stress models, where pre-dispositional vulnerabilities and intense exposure heighten the risk of severe psychological responses [28, 32]. Trauma proximity and intensity often override baseline defenses, contributing to PTSD [33], underscoring the need for interventions targeting cumulative stress and promoting resilience.

#### Trauma recovery trajectory

Interestingly, 9.4% of participants with high pre-attack PTSD severity showed significant symptom reduction after the attack, suggesting that collective trauma can, paradoxically, aid recovery. This is particularly interesting because pre-existing symptoms refer to pre-attack traumatic events, and post-attack symptoms refer to the attack. This may align with the “trauma coherence hypothesis,” where external threats validate internal struggles, fostering psychological integration [34]. Shared national trauma might also provide solidarity and validation, aiding recovery. Alternatively, acute survival demands may have activated coping strategies, temporarily alleviating pre-existing symptoms. Future studies are needed to more fully understand the complex interplay between past trauma and acute stress in shaping outcomes.

### Trauma exposure and trajectories of PTSD

The study highlighted predictors of PTSD trajectories, showing the interplay of individual predispositions, social contexts, and trauma characteristics. Younger individuals were overrepresented in the trauma vulnerability and chronic PTSD trajectories, likely due to fewer coping mechanisms and less life experience [35]. Relationship status was associated with outcomes, with those without a partner more likely to fall into these trajectories, emphasizing the protective role of social support [36]. Supportive relationships likely provide emotional stability and security during crises.

Current (war) trauma exposure intensity was a key factor differentiating trajectories, with participants in the trauma vulnerability and recovery groups reporting higher exposure to war-related stressors, such as direct threats, frequent sirens, and graphic media coverage. These findings align with evidence that trauma proximity and severity drive PTSD symptoms [33], with the October 7 attack’s graphic documentation likely amplifying stress responses in vulnerable individuals.

Cumulative trauma history, spanning childhood and adulthood, distinguished PTSD trajectories. Childhood abuse and stress were prominent in the chronic PTSD group, suggesting early adversity disrupts development and stress-response systems. In the trauma vulnerability group, adulthood trauma and accumulated stressors were more significant. These findings highlight how early trauma increases sensitivity to later stressors [28].

The trauma recovery trajectory, marked by symptom improvement post-attack, is associated with high childhood abuse and adult trauma levels as well as war-related stressors. This is surprising given that trauma-vulnerability had similar correlations with traumatic events during life, and indeed, both groups responded to the October 7 attack, but in a different manner.

Although gender differences were not significant in this study, they merit further investigation. Research suggests women may be more prone to PTSD due to socialization, neurobiology, and exposure patterns [37]. Future studies should explore how gender interacts with other factors to shape PTSD trajectories.

### Clinical and policy implications

Distinct PTSD trajectories require tailored interventions. For the trauma vulnerability group, early sustained support, including psychoeducation, resilience-building, and trauma-focused therapies, is essential. Chronic PTSD requires addressing lifetime trauma through therapies like prolonged exposure and EMDR. Symptom reduction in trauma recovery merits study to understand how collective trauma fosters recovery, potentially informing therapies leveraging shared experiences. While the resilience group likely faced fewer traumatic events, some remained resilient despite significant stressors, highlighting factors beyond reduced trauma. Findings emphasize targeted interventions and suggest machine learning to refine PTSD trajectory prediction.

### Study limitations

This study’s strengths include an approximately nationwide Israeli Jewish sample, a longitudinal design, and pre-trauma data. However, limitations remain. First, restricting participation to Jewish, Hebrew-speaking individuals excludes approximately 25% of Israeli citizens (mainly Arab citizens), limiting generalizability across Israel’s diverse population. Online panel recruitment may introduce selection bias, as voluntary participants may differ systematically from the general population. The quasi-representative sampling cannot account for all characteristics affecting PTSD trajectories, and findings may not generalize beyond this specific population and trauma context. Second, the year before the October 7 attack involved political polarization and protests, raising questions about whether T3 symptom changes resulted solely from the attack and war or were influenced by prior unrest. T3 PTSD captures attack- and war-related trauma, but political unrest between T2 and T3 may also be a risk factor, warranting further study of both contexts. Third, self-reported measures may introduce recall bias, and variations in stressor type and duration were not fully addressed. Fourth, dropouts between measurements may bias results, despite efforts to handle missing data. Finally, the absence of genetic and neurobiological data limits insights into underlying mechanisms.

## Conclusions

This study highlights diverse PTSD trajectories following collective trauma, with most civilians showing resilience while others experienced chronic distress, vulnerability, or recovery. These findings emphasize the need for personalized interventions addressing individual and contextual factors, contributing to strategies that mitigate long-term impacts of collective trauma.

## Supporting information

10.1192/j.eurpsy.2025.10130.sm001 Levin et al. supplementary material Levin et al. supplementary material

## Data Availability

De-identified data underlying this article will be made available by the corresponding author upon reasonable request for research purposes, subject to institutional approvals and a data use agreement.

## Supplementary material

The supplementary material for this article can be found at http://doi.org/10.1192/j.eurpsy.2025.10130.
