# Supplementary material for: Longitudinal PTSD trajectories before and after the October 7, 2023, terror attacks: A nationwide study of Israeli adults
Source: Eur Psychiatry. 2025 Nov 3;69(1):e13. doi: 10.1192/j.eurpsy.2025.10130 (PMC12925667; doi:10.1192/j.eurpsy.2025.10130)
Supplement: Levin et al. supplementary material [file S0924933825101302sup001.docx]

**Supplementary materials**

**The association between schizophrenia-related negative symptoms and complex posttraumatic stress disorder: A transdiagnostic approach**

**Statistical Analyses**

The normality of continuous variables was tested by Kolmogorov-Smirnov test, Q-Q plots, and skewness. Since the SNS variables had non-normal distribution, the interquartile ranges and medians are presented in addition to the means ±standard deviations (SD). Univariate analyses of variance (ANOVA) were used to determine the preliminary differences between groups of CPTSD, PTSD, and no disorder in scores of SNS total score and each of the subscales.

**Results**

Kolmogorov-Smirnov tests for normality of the SNS subscales were significant, indicating that the observed data significantly deviates from a normal distribution (Statistics ranged from .120 of the blunted affect to .272 of the anhedonia subscales and all p values < .001). This is in accordance with the Skewness which ranged from .218 to .959 with Std values of .06, and the anhedonia scale which was shown to be more skewed (Skewness = 1.40 Std = .06). This points to a distribution that is positively skewed, with the tail of the distribution extends to the right, as expected.

The SNS scale mean score was 10.63 (SD = 7.59) with a range of 0-40. The SNS interquartile range was 5-16) along with each subscale Median and mean scores (SD) starting with asociality: Med = 2.00 M = 2.29 (2.07) interquartile range: 1-4, blunted affect: Med = 3.00 M = 2.73 (1.70) interquartile range: 2-4, alogia: Med = 1.00 M = 1.96 (2.14) interquartile range: 0-4, avolition: Med = 2.00 M = 2.34 (2.21) interquartile range: 0-4, and anhedonia: Med = 1.00 M = 1.31 (1.74) interquartile range: 0-2.

Initial examination of the group differences in the SNS scores (see table SM1 in supplementary materials) revealed that the total SNS score and asociality, alogia, avolition, and anhedonia were significantly different between the group with the post hoc analyses revealing specific patterns according to CPTSD group reported higher SNS compared to both PTSD and the no disorder groups (table 1 shows the means and standard deviations with different uppercase letters representing the differences between the groups). As for the blunted affect, the differences were significant with post hoc analysis revealing differences between all groups. CPTSD participants reported higher blunted affect than PTSD and no disorder, but also PTSD reported higher blunted affect compared to the no disorder group.

Table SM1. STROBE Statement—Checklist of items that should be included in reports of cross-sectional studies

|  | **Item No** | **Recommendation** |  |
| --- | --- | --- | --- |
| **Title and abstract** | 1 | (*a*) Indicate the study’s design with a commonly used term in the title or the abstract | V |
|  |  | (*b*) Provide in the abstract an informative and balanced summary of what was done and what was found | V |
| **Introduction** | | |  |
| Background/rationale | 2 | Explain the scientific background and rationale for the investigation being reported | V |
| Objectives | 3 | State specific objectives, including any prespecified hypotheses | V |
| **Methods** | | |  |
| Study design | 4 | Present key elements of study design early in the paper | V |
| Setting | 5 | Describe the setting, locations, and relevant dates, including periods of recruitment, exposure, follow-up, and data collection | V |
| Participants | 6 | (*a*) Give the eligibility criteria, and the sources and methods of selection of participants | V |
| Variables | 7 | Clearly define all outcomes, exposures, predictors, potential confounders, and effect modifiers. Give diagnostic criteria, if applicable | V |
| Data sources/ measurement | 8* | For each variable of interest, give sources of data and details of methods of assessment (measurement). Describe comparability of assessment methods if there is more than one group | *V* |
| Bias | 9 | Describe any efforts to address potential sources of bias | V |
| Study size | 10 | Explain how the study size was arrived at | V |
| Quantitative variables | 11 | Explain how quantitative variables were handled in the analyses. If applicable, describe which groupings were chosen and why | V |
| Statistical methods | 12 | (*a*) Describe all statistical methods, including those used to control for confounding | V |
|  |  | (*b*) Describe any methods used to examine subgroups and interactions | V |
|  |  | (*c*) Explain how missing data were addressed | V |
|  |  | (*d*) If applicable, describe analytical methods taking account of sampling strategy | V |
|  |  | (*e*) Describe any sensitivity analyses | V |
| **Results** | | |  |
| Participants | 13* | (a) Report numbers of individuals at each stage of study—eg numbers potentially eligible, examined for eligibility, confirmed eligible, included in the study, completing follow-up, and analysed | V |
|  |  | (b) Give reasons for non-participation at each stage | V |
|  |  | (c) Consider use of a flow diagram |  |
| Descriptive data | 14* | (a) Give characteristics of study participants (eg demographic, clinical, social) and information on exposures and potential confounders | V |
|  |  | (b) Indicate number of participants with missing data for each variable of interest |  |
| Outcome data | 15* | Report numbers of outcome events or summary measures | V |
| Main results | 16 | (*a*) Give unadjusted estimates and, if applicable, confounder-adjusted estimates and their precision (eg, 95% confidence interval). Make clear which confounders were adjusted for and why they were included | V |
|  |  | (*b*) Report category boundaries when continuous variables were categorized | V |
|  |  | (*c*) If relevant, consider translating estimates of relative risk into absolute risk for a meaningful time period | V |
| Other analyses | 17 | Report other analyses done—eg analyses of subgroups and interactions, and sensitivity analyses | V |
| **Discussion** | | |  |
| Key results | 18 | Summarise key results with reference to study objectives | V |
| Limitations | 19 | Discuss limitations of the study, taking into account sources of potential bias or imprecision. Discuss both direction and magnitude of any potential bias | V |
| Interpretation | 20 | Give a cautious overall interpretation of results considering objectives, limitations, multiplicity of analyses, results from similar studies, and other relevant evidence | V |
| Generalisability | 21 | Discuss the generalisability (external validity) of the study results | V |
| **Other information** | | |  |
| Funding | 22 | Give the source of funding and the role of the funders for the present study and, if applicable, for the original study on which the present article is based | V |

Table SM2. Univariate analysis of variance for group differences in the SNS factors

|  | CPTSD | PTSD | No disorder | F (2, 1813) |
| --- | --- | --- | --- | --- |
| SNS total | 15.05 (7.71)^a^ | 8.30 (5.95)^b^ | 8.34 (6.43)^b^ | 193.11*** |
| Asociality | 3.31 (2.14)^a^ | 1.92 (1.89)^b^ | 1.74 (1.81)^b^ | 129.57*** |
| Blunted affect | 3.21 (1.61)^a^ | 2.82 (1.54)^b^ | 2.45 (1.68)^c^ | 41.91*** |
| Alogia | 2.84 (2.28)^a^ | 1.40 (1.75)^b^ | 1.52 (1.92)^b^ | 91.19*** |
| Avolition | 3.46 (2.21)^a^ | 1.84 (1.92)^b^ | 1.81 (2.01)^b^ | 131.61*** |
| Anhedonia | 2.22 (2.03)^a^ | .92 (1.31)^b^ | .82 (1.33)^b^ | 153.25*** |

Notes. ***p<0.001. different uppercase letters represent significant group differences
